# The Interplay of Quality of Life and Psychological Distress Among Egyptian Migrants in Australia: A Cross-Sectional Study

**DOI:** 10.3390/healthcare13222853

**Published:** 2025-11-10

**Authors:** Gihane Endrawes, Wenpeng You

**Affiliations:** 1 School of Nursing and Midwifery, Western Sydney University, Locked Bag 1797, Penrith, NSW 2751, Australia; w.you@westernsydney.edu.au; 2 Adelaide Medical School, The University of Adelaide, Frome Road, Adelaide, SA 5005, Australia

**Keywords:** quality of life, psychological distress, Egyptian migrants, mental well-being, cross-cultural health, Kessler scale

## Abstract

Background: The interplay between QoL and psychological distress may differ cross-culturally. The purpose of this study was to investigate the relationship between quality of life (QoL) and psychological distress, among an Egyptian Christian background sample. Methods: Participants completed QoL and K-10 questionnaires. Descriptive statistics, correlation analysis, and partial correlation controlling for age were conducted to explore these relationships. Results: The sample had a mean age of 50.64 years (SD ± 9.6) and was slightly male dominated (57.1%). QoL scores ranged from 4.23 to 5.52 on a 7-point scale, with the highest scores in personal relationships and the lowest in community engagement. K-10 scores indicated low to moderate psychological distress, with feeling tired without reason scoring highest. A significant negative correlation was found between QoL and K-10 scores (r = −0.354, *p* < 0.001), suggesting higher QoL is associated with lower psychological distress. Material comforts and health showed the strongest negative correlations with K-10 scores. The relationship between QoL and psychological distress remained significant after controlling for age (r = −0.347, *p* < 0.01). Self-awareness and self-expression emerged as key factors positively correlated with overall QoL. Conclusions: Enhancing QoL, particularly in areas of material comfort, health, and personal growth, may effectively reduce psychological distress. Interventions should be culturally tailored to respect linguistic and religious backgrounds. Further research with more diverse samples and longitudinal designs is recommended to deepen understanding of these relationships.

## 1. Introduction

The concept of quality of life (QoL) has received increasing attention in both academic discourse and healthcare practice [1,2]. Defined as an individual’s perception of their position in life within the context of culture and value systems in which they live, QoL encompasses various domains including physical health, psychological state, social relationships, and environmental factors [3,4]. Concurrently, mental health remains a fundamental component of overall well-being, influencing cognitive, emotional, and behavioral aspects of individuals [5,6]. The intricate interplay between these two constructs, QoL and mental health, has become a focal point of research, particularly in understanding how improvements in one domain can positively impact the other [7,8].

The relationship between QoL and mental health is bidirectional and complex [9]. Individuals experiencing mental health challenges often report lower subjective well-being and perceived QoL due to impaired functioning and reduced satisfaction across multiple life domains [10]. Conversely, higher levels of QoL have been associated with better mental health outcomes [11]. Research indicates that individuals who perceive their life circumstances favorably and report higher levels of satisfaction experience lower levels of psychological distress, enhanced resilience, and improved coping mechanisms [12,13].

Moreover, the determinants of both QoL and mental health are multifaceted. Individual factors such as socio-demographic characteristics (e.g., age, gender, socioeconomic status), health status, and personal experiences play crucial roles in shaping an individual’s perceived QoL and mental health [14,15]. Contextual factors including social support networks, economic stability, access to healthcare services, and environmental conditions also significantly influence these constructs [16,17]. Recognizing and addressing the factors influencing mental health and QoL is essential for fostering holistic well-being. Effective strategies include enhancing social support systems, improving access to mental health services, reducing social inequalities, and fostering community engagement [1,18]. Additionally, creating supportive environments can build resilience and lead to better mental health outcomes across diverse populations. [18,19].

The interdependence of QoL and mental health highlights the need for integrated and comprehensive approaches to healthcare and well-being [20]. By addressing the factors that contribute to both constructs, researchers and practitioners can work towards enhancing the overall QoL and mental health outcomes for individuals and communities alike [21,22]. This study aimed to contribute to the existing literature by exploring the nuanced relationship between QoL and psychological distress within an Egyptian Christian community. This population is uniquely positioned within a distinct cultural and religious context, which influences their experiences of both QoL and psychological stress. By examining this specific community, the study seeks to elucidate the cultural and contextual factors that shape these constructs and their interrelation. Understanding these dynamics is crucial for developing culturally sensitive strategies that can effectively enhance QoL and reduce psychological distress.

Quality of life (QoL) and psychological distress are key concerns for migrants, who often face disrupted social networks, cultural adaptation challenges, and barriers to healthcare access. These stressors can negatively affect well-being, with lower QoL frequently linked to higher psychological distress. While community-based and culturally tailored interventions in Australia aim to address these issues, their reach has been limited. Recent evidence highlights the promise of digital interventions; for example, Ng et al. showed that accessibility, cultural sensitivity, and engagement strategies are vital for improving migrant well-being and reducing distress [23]. Nonetheless, research gaps remain in understanding how specific sociocultural characteristics influence QoL and mental health outcomes. This study contributes to filling this gap by focusing on Egyptian migrants in Australia, a community with distinct cultural and religious features.

## 2. Methods

QoL was assessed using the Flanagan Quality of Life Scale [24], which included 16 items across three sub-domains: (1) Relationships and Material well-being, (2) Personal Social and Community Commitment, and (3) Health and Functioning. The K-10 consists of 10 items with a response format capturing the frequency of symptoms over a four-week period. Both scales emphasize the need for culturally and linguistically sensitive tools, with the K-10 translated into Arabic and validated among Arabic-speaking populations, and the Flanagan QoL scale demonstrating reliability and validity in culturally diverse groups.

### 2.1. Study Design

The study was cross-sectional and targeted migrant Egyptians. Eligibility criteria included: (1) Attendance at one of three local Egyptian Coptic churches in Sydney, Australia; (2) Age 18 years or older; (3) No impairment in comprehension due to diagnosed mental health conditions or dementia; and (4) Ability to read in either English or Arabic. The church setting was selected due to its significant influence on lifestyle and health behaviors. Demographic factors such as age, sex, marital status, employment duration, employment type, retirement status, and reason for retirement are considered as they impact QoL and mental health. Psychological distress was measured using the K10 scale. The study included additional questions beyond the 26 scaled items, covering anticipated changes in social life, spiritual life, physical activity, and other areas, as well as an open-ended question on concerns impacting QoL. Participants were also asked about their ability to function and the impact of mental health issues on their daily activities. Four multiple-choice questions address participants’ understanding of the frequency, duration, and impact of health-related feelings on their functioning and interaction with healthcare [25].

### 2.2. Pilot Testing

All 34 questions were pilot-tested with a convenience sample of five Arabic-speaking adults attending church services at one of the three study sites. One-on-one cognitive interviews employing think-aloud and probing techniques were conducted to evaluate participants’ understanding and interpretation of the questions [26]. Following item generation, a panel of six experts, including mental health nursing clinicians and academic researchers, evaluated the relevance of each of the 32 items using a 4-point scale from 1 (not relevant) to 4 (very relevant). Content validity was determined based on the proportion of experts rating an item as 3 or 4. All 34 items achieved an average relevance score of 0.82 or higher and were retained for the study.

### 2.3. Participant and Sampling

This study followed established protocols for research involving human subjects and received approval from the University Human Research Ethics Committee. The cross-sectional study was carried out at three local Egyptian Coptic churches in Sydney, Australia. Churches have been commonly utilized in previous research due to their significant role in fostering health-related behaviour changes, including smoking cessation, breast cancer screening, diabetes prevention, nutrition, and physical activity within diverse communities [27,28]. The researcher collaborated with church leaders to explain the study’s objectives and gain their support for distributing questionnaires. The leaders announced the study to the community during services, while the bilingual first author attended services to engage with attendees, share information, and address any questions. For scale testing and six associated items, the minimum sample size was calculated using a ratio of 5 participants per item [29,30]. To account for a potential 20% rate of invalid or missing responses, a sample size of 200 participants was deemed adequate. Consequently, a total of 200 questionnaires were distributed, and 156 valid responses were included in the final analysis. The remaining 44 were excluded due to incomplete data or failure to meet inclusion criteria. Based on Cohen’s (1988) guidelines for detecting moderate correlations (r ≈ 0.30) with α = 0.05, the final sample size yields a statistical power of approximately 0.96, which is considered highly adequate for the study’s objectives [31]. This exceeds the commonly accepted threshold of 0.80, indicating that the study was well-powered to detect meaningful associations between the QoL and K-10 measures.

Minor variations in N across tables reflect occasional item-level non-responses (e.g., skipped or ‘not applicable’ items), which were handled using pairwise deletion to preserve valid cases without affecting the total sample size (N = 156) or statistical power.

### 2.4. Data Analysis

Descriptive statistics were used to summarize the self-reported demographic characteristics of the sample. For the 26 scaled questions, descriptive statistics included the minimum, maximum, mean, standard deviation, and sample size. Scatter plots were created to examine the correlation between the mean scores of QoL and the K-10 scales and assess the data quality. For each participant, mean scores were computed for all 16 QoL questions and all 10 K-10 questions, generating two new variables: QoL mean and K-10 mean. The four additional questions included in the K10+ assessment do not influence the K-10 score but are intended to reflect the impact of psychological distress on daily life. Consequently, the mean K-10 score, derived from the 10 questions of the K-10 scale, remains unchanged.

Correlation coefficients between these means and the 26 individual scaled questions (totalling 28 items/variables) were computed using a non-parametric model. Since all demographic factors except age are categorical or nominal, age was considered a confounding variable in the analysis of the correlation coefficients between the 28 items. Logistic regression analyses were conducted to identify demographic factors associated with the well-being of Egyptian migrants, such as age, gender, and type of employment. This analysis helped to determine if QoL and K-10 scores were related to these demographic factors. For non-scaled questions, the frequencies of ‘Yes’ and ‘No’ responses were counted for three closed questions: Do you have any plans or expect any changes on the following areas after retirement from employment? Physical activity, spiritual and social life? Do you have any plans or expect any changes on other areas after retirement from employment? Do you have any concerns impacting on your life quality? Mean scores were calculated for each of the multiple-choice questions, and responses to the single open-ended question were analysed. In addition to the 26 scaled questions, all other questions, including those four K-10 associated additional questions, were reported and associated with the statistical analysis results to discuss the relationship between QoL and K-10.

## 3. Results

### 3.1. Demographic Factor Descriptives

Table 1 presents the sample characteristics of 156 participants in the study. The key findings include:

The sample has a mean age of 50.64 years (SD ± 9.6), with ages ranging from 28 to 73. There is a slight majority of participants over 50 (n = 89) compared to those 50 or younger (n = 66). The gender distribution shows more males (n = 89) than females (n = 67). Most respondents completed the questionnaire in Arabic (n = 98) rather than English (n = 59). The sample is predominantly Christian (n = 155) and of Egyptian ethnicity (n = 137). The majority are living with a partner (n = 146) and employed (n = 154). Among those employed, most are in professional or semi-professional roles (n = 83), with fewer in skilled or self-employed positions (n = 37). Among the 154 employed participants, professional and semi-professional roles included doctors, nurses, teachers, and social workers, while skilled and self-employed roles comprised labourers, tradespeople, and small business owners. This distribution indicates a generally stable socio-economic profile, which may partly explain the participants’ higher quality of life and lower psychological distress scores. While most participants are still working, some have retired, with retirement durations ranging from 0 to 9 years (n = 12) to 10–20+ years (n = 17). Common reasons for retirement include being tired and age (n = 12), illness (n = 11), and family reasons (n = 5). This demographic profile suggests a sample of predominantly middle-aged, employed, partnered Egyptian Christians, with a mix of language preferences and professional backgrounds.

### 3.2. Data Descriptive

Table 2 presents descriptive statistics for 26 survey questions covering QoL and psychological distress (K-10). QoL questions used a 7-point scale, with most means between 5 and 5.5, indicating generally positive life experiences. Close relationships scored highest (mean 5.52), while participation in organizations scored lowest (mean 4.23). The question about having children showed the most variability. K-10 questions primarily used a 5-point scale, with means ranging from 1.8 to 2.7, suggesting low psychological distress levels. Feeling tired without reason was the most common (mean 2.75), while feeling worthless was the least reported (mean 1.78). K-10 responses showed less variability than QoL responses. Overall, respondents reported positive QoL with some variation, and consistently low psychological distress levels. These findings offer insights into the population’s well-being, highlighting strengths in personal relationships.

### 3.3. Scatter Plots

**Figure 1:**
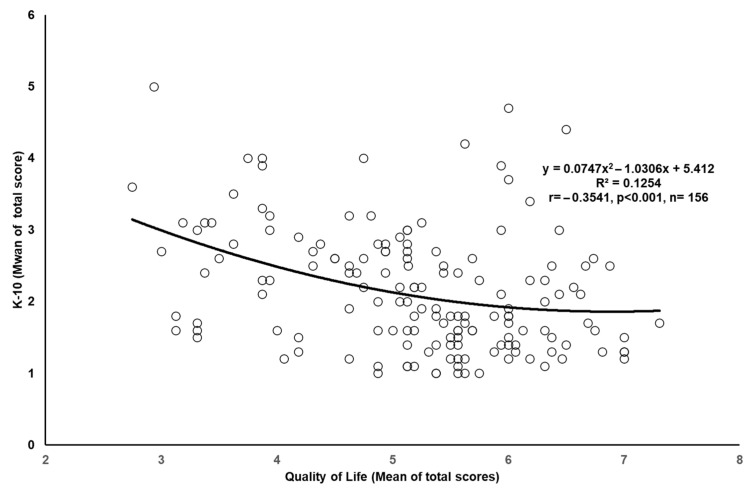
Correlation between quality-of-life score and mental health score.

Figure 1 showed a polynomial correlation between QoL and K-10 scores, represented by the equation y = 0.0747x^2^ − 1.0306x + 5.412 (R^2^ = 0.1254). Analysis revealed a significant negative correlation between QoL and K-10 means (r = −0.354, *p* < 0.001, n = 156). This indicates that higher QoL scores are associated with lower K-10 scores (indicating lower psychological stress). The scatter plot illustrates this relationship, with a polynomial correlation equation (y = 0.0747x^2^ − 1.0306x + 5.412) and an R^2^ value of 0.1254, showing a moderate degree of fit.

### 3.4. Non-Parametric Correlation

Table 3 reveals that the correlation analysis presented a nuanced relationship between QoL and psychological distress. Overall, higher QoL scores were consistently linked to lower psychological distress, demonstrating a significant negative correlation. Within the QoL domain, all aspects of life quality show positive interconnections, highlighting the holistic nature of well-being. Notably, understanding of oneself emerged as a central factor, showing the strongest link to overall QoL. This underscores the importance of self-awareness and personal growth in fostering life satisfaction. The K-10 scale showed strong internal consistency, with all items closely interrelated. Persistent low mood emerged as a significant contributor to psychological distress. A negative correlation was observed between QoL and K-10 measures, with material comforts consistently linked to lower psychological distress, highlighting the potential buffering effect of financial security and physical comfort on mental health.

However, the analysis revealed that some aspects of QoL, such as participation in organizations and independence, had weaker or non-significant relationships with psychological distress. This complexity in the data reminds us that the relationship between QoL and mental health is multifaceted, with different life domains influencing psychological well-being to varying degrees.

### 3.5. Partial Correlation Exploring the QoL-K10 Relationship Independent of Age

Table 4 presents a partial correlation analysis examining the relationship between QoL measures and the K-10 psychological distress scale, while controlling for age. This analysis revealed insights into how these variables interacted independently of age-related influences. The overall negative correlation between mean QoL and mean K-10 scores (r = −0.347, *p* < 0.01) persisted when controlling for age, indicating that higher QoL was associated with lower psychological distress across age groups. Within the QoL domain, all aspects maintained positive intercorrelations, suggesting that various QoL factors were interrelated regardless of age. Notably, QoL12 (Expressing yourself creatively, r = 0.755, *p* < 0.01) and QoL 10 (Understanding yourself, r = 0.743, *p* < 0.01) show the strongest correlations with mean QoL. This underscores the importance of self-expression and self-understanding in overall QoL, independent of age. The K-10 items continued to demonstrate strong positive correlations with each other and with the mean K-10 score, reflecting the internal consistency of the psychological distress measure across age groups. K-10, 9 (feeling so sad that nothing could cheer you up) maintains the strongest correlation with mean K-10 (r = 0.871, *p* < 0.01), emphasizing the central role of persistent sadness in overall psychological distress. Examining the relationships between QoL and K-10 items, most correlations remained negative and significant after controlling for age, though their strengths varied. Work (QoL 11) exhibited the strongest negative correlation with mean K-10 (r = −0.428, *p* < 0.01), suggesting that work satisfaction was particularly important for mental health across different age groups. However, QoL aspects like having and rearing children (QoL 4) and independence (QoL 16) displayed weaker or non-significant correlations with K-10, suggesting their influence on psychological distress may depend more heavily on age-related factors. This highlights the complexity of how specific life domains interact with mental health.

### 3.6. Logistic Regression

Table 5 presents the logistic regression analysis, which revealed intriguing relationships between demographic factors, QoL, and psychological distress. Age, categorized as 50 years and younger versus over 50 years, showed no statistically significant relationship with either QoL or K10. However, age accounts for 14.8% of the variance in QoL and 12.2% in K-10, suggesting a potential influence. The language used for the survey, whether English or Arabic, did not show statistical significance for QoL or K-10, but it explained 34.2% of the variance in QoL, hinting at a possible role in perceptions of quality of life. Gender differences between males and females were not statistically significant for QoL or K-10. The gender model accounts for 15.8% of the variance in QoL but only 4.4% in K-10. Employment status (full-time versus part-time/retired) showed no statistical significance for QoL or K-10, explaining 11.2% and 6.1% of the variance, respectively. The most notable finding was the significant relationship between employment type and QoL (*p* = 0.034). This model explained 29.2% of the variance in QoL, highlighting a meaningful connection between job type and perceived QoL. However, this relationship was not significant for K-10, explaining 11.4% of the variance.

For questions not measured with scales, participants were asked about their plans or expected changes after retirement in three areas: social life (Yes = 96, No = 16), spiritual life (Yes = 115, No = 4), and physical activity (Yes = 115, No = 0). An open-ended question revealed intentions to engage in more physical activities, travel, run a business, volunteer, and tutoring. Another open-ended question asked, “Do you have any concerns impacting your quality of life?” Responses included adjusting to a new culture, financial concerns, social life constraints, reduced church participation, chronic diseases, and decreased family time. The last 4 multiple-choice questions from the K-10+ were asked about the impact of feelings on daily activities, healthcare visits, and physical health problems over the last 30 days. The mean scores were: 1.29 days totally unable to function, 1.73 days with reduced activities, 1.32 healthcare visits, and 1.83 times physical health problems were the main cause of these feelings.

While many studies use established cut-off points to categorize QoL and K-10 scores for clinical interpretation, we deliberately chose item-level analysis in this study. This approach was adopted to capture nuanced variation across domains, given the distinct cultural and religious context of Egyptian migrants. Predetermined thresholds, often derived from other populations, may overlook culturally specific patterns. By examining items individually, we were able to identify domains such as material comfort, health, and self-awareness that were particularly salient in this cohort. This strategy also reflects the exploratory nature of the study, providing a culturally sensitive baseline to inform future intervention research. In subsequent studies, item-level analysis may be complemented with cut-off-based approaches to enhance clinical comparability.

## 4. Discussion

The present study investigated the relationship between QoL and psychological distress, as measured by the Kessler Psychological Distress Scale (K-10) among 156 predominantly middle-aged, employed, partnered Egyptian Christians. Our findings provide significant insights into how these variables interact and highlight important demographic influences. Simple regressions (scatter plot and non-parametric correlation) demonstrate a clear trend: as QoL scores increase, K-10 scores decrease, indicating reduced psychological distress. Similar studies have reported analogous findings. For instance, research by Westerhof et al. found a significant negative correlation between QoL and psychological distress, highlighting that better QoL reduces the likelihood of experiencing high levels of psychological distress [32]. This study also emphasized the importance of physical health and social support in enhancing overall QoL and mitigating mental health issues. Additionally, a study by Lamers et al. reported that individuals with higher life satisfaction and stronger social relationships tend to have lower levels of psychological distress [33]. The study emphasized that social relationships provide emotional support, which can significantly buffer against the effects of stress and improve mental health. Furthermore, the World Health Organization’s Quality of Life assessment (WHOQOL) consistently shows that individuals with better physical health, social relationships, and environmental conditions report lower levels of psychological distress, supporting the idea that a higher QoL can protect against mental health problems [34,35,36].

The significant negative correlation between QoL and psychological distress aligns with global findings [37,38], suggesting that core QoL aspects such as physical health, emotional well-being, and social relationships universally influence psychological distress across cultures [39,40]. The polynomial correlation equation identified in the scatter plot provides a more nuanced understanding of this relationship. The R^2^ value of 0.1254 suggests a moderate fit and the existence of additional factors influencing psychological distress. Nevertheless, the clear negative correlation reinforces the importance of improving QoL in mental health interventions.

The demographic characteristics of the sample offer valuable context highlighting how high QoL reduces psychological distress among Arabic-speaking participants, aligning with existing literature. While religious faith generally supports maintaining healthy lifestyles and QoL, certain characteristics of this cohort are particularly noteworthy for their beneficial effects on health and QoL [41,42]. Arabic-speaking populations may have cultural tendencies that influence how psychological distress is experienced and reported. Cultural norms and values significantly impact mental health perceptions and expressions [43]. For instance, in many Arabic cultures, there is a strong emphasis on spirituality, social support, family ties, and community cohesion, which can enhance QoL and provide a buffer against psychological distress [44]. Arabic-speaking individuals might have developed specific resilience and coping mechanisms that mitigate psychological distress. For example, religious and spiritual practices common in many Arabic-speaking countries have been shown to provide significant mental health benefits, offering comfort and a sense of purpose during stressful times [45,46].

The importance of strong social support networks, often prevalent in Arabic-speaking communities, could contribute to the observed significant relationship [47]. Research indicates that social support is crucial in mental health, helping individuals manage stress and enhancing overall life satisfaction [40]. This social support might be more accessible and utilized within close-knit Arabic-speaking communities, leading to a stronger correlation between high QoL and low psychological distress. In many Middle Eastern cultures, strong family ties and community bonds are highly valued [47]. The study reveals significant negative correlations between close personal relationships (e.g., with a spouse or significant other) and psychological distress. This aligns with existing literature that emphasize the protective effects of strong social support networks on mental health. Strong interpersonal relationships provide emotional support, reduce feelings of isolation, and enhance an individual’s ability to cope with stressors [48]. For instance, married individuals often report better mental health outcomes compared to their unmarried counterparts, possibly due to the emotional and practical support that a partner provides. Descriptive statistics indicate generally positive QoL among participants, with scores ranging from 4.23 to 5.52 on a 7-point scale. The highest mean scores were observed in the domain of close relationships with a spouse or significant other (mean 5.52), highlighting the importance of personal relationships in QoL.

However, our analysis is more in-depth while age has been considered the important factor impacting QoL and mental health, our partial correlation analysis controlling for age revealed that the overall negative correlation between mean QoL and mean K-10 scores (r = −0.347, *p* < 0.01) remained significant when age was accounted for [49]. This indicates that the relationship between QoL and psychological distress is consistent across different age groups, suggesting that interventions aimed at improving QoL could be broadly applicable. Notably, self-expression (QoL 12) and self-understanding (QoL 10) had the strongest correlations with mean QoL, emphasizing the enduring importance of self-expression and self-awareness in enhancing life quality [50]. This analysis suggests the potential benefits of comprehensive strategies that address multiple facets of an individual’s life to enhance overall well-being and reduce psychological distress [51]. For instance, interventions that improve physical health, strengthen social support networks, and promote meaningful activities can collectively contribute to better mental health outcomes. As such, these findings reinforce the need for holistic approaches in both clinical and community settings to effectively support mental health and enhance QoL.

The cultural characteristics of the 156 Arabic-speaking participants illustrate how high QoL can reduce psychological distress. Analysis of demographic factors shows that these variables did not significantly correlate with QoL or psychological distress overall. Logistic regression analysis provided insights into these factors’ effects on QoL and psychological distress, measured by K-10. Age (50 years and younger vs. over 50) did not significantly relate to QoL or K-10, though it accounted for 14.8% of the variance in QoL and 12.2% in K-10, suggesting potential relevance. Language preference (English vs. Arabic) did not significantly affect QoL or K-10 but explained 34.2% of the variance in QoL, hinting at its potential impact on life perceptions. Gender differences did not show statistical significance, with the gender model explaining 15.8% of the variance in QoL and 4.4% in K-10. Employment status (full-time vs. part-time/retired) also had no significant impact on QoL or K-10, explaining 11.2% and 6.1% of the variance, respectively. Marital status did not significantly impact QoL or K-10, accounting for 11.3% and 19.8% of the variance, respectively. The high proportion of participants living with a partner (93.6%) may explain the lack of significant findings. A notable result was the significant relationship between employment type and QoL (*p* = 0.034), with the model explaining 29.2% of the variance. This indicates a meaningful link between job type and QoL, though not significant for K-10, which explained 11.4% of the variance. The homogeneity among participants—such as those being predominantly middle-aged, employed, partnered Egyptian Christians with shared culture, employed in professional or semi-professional roles, and religious beliefs—could impact these findings. This highlights the importance of considering diverse factors in mental health research to gain a comprehensive understanding of QoL and psychological distress.

Our regression analysis revealed a significant negative association between quality of life and psychological distress, highlighting that higher QoL reduces the likelihood of experiencing elevated distress. This finding aligns with previous research that has demonstrated similar patterns across diverse populations. For instance, Westerhof and Keyes reported that higher levels of well-being and life satisfaction were consistently linked with lower psychological distress across the lifespan [32]. Similarly, Lamers et al. found that life satisfaction and strong social relationships served as protective factors against psychological distress, enhancing overall mental health outcomes [33]. Studies based on the WHOQOL framework further confirm that improved physical health, strong social support networks, and stable environmental conditions act as buffers against psychological distress [34,35]. These consistencies reinforce the robustness of our findings and suggest that the relationship between QoL and psychological distress observed among Egyptian migrants is consistent with broader international evidence.

The findings should also be interpreted within the cultural and contextual background of the participants. The Egyptian Christian community places strong emphasis on faith, family cohesion, and mutual social support, which may buffer against psychological distress and contribute to higher quality of life. Religious coping, community engagement through the church, and culturally rooted values such as gratitude and resilience likely play a role in maintaining well-being despite migration-related challenges. These cultural and contextual influences align with the study’s aim to elucidate the factors shaping the relationship between QoL and psychological distress in this population.

## 5. Conclusions

This study highlighted the significant relationship between QoL and psychological distress, and the importance of holistic well-being. Improving material comforts, physical health, personal relationships, and self-awareness can help reduce psychological distress. Culturally tailored interventions that honor the linguistic and religious backgrounds of individuals are essential. The study’s findings offer practical implications for healthcare providers and policymakers. The negative correlation between QoL and psychological distress suggests that improving QoL could alleviate psychological distress. This might involve programs aimed at enhancing material comfort, physical health, and personal relationships. Additionally, the strong link between self-awareness and overall QoL indicates that fostering self-reflection and personal growth through counseling, workshops, and community programs could significantly benefit well-being. While this study provides valuable insights, it has limitations that should be addressed in future research The sample was predominantly middle-aged, employed, partnered Egyptian Christians, which limits the generalizability of the findings to other populations. Future studies should aim to include more diverse samples in terms of age, employment status, marital status, cultural background, and pre-migration status. Further studies are needed to explore how migration pathways influence psychological distress and quality of life, particularly among underrepresented CALD groups, to differentiate between forced and planned migrants to better understand and address mental health and quality of life issues in a culturally sensitive manner. Additionally, the cross-sectional design of the study limits the ability to infer causality between QoL and psychological distress. Longitudinal studies are needed to examine how changes in QoL over time affect psychological well-being. Furthermore, qualitative research could provide deeper insights into the subjective experiences of participants, offering a richer understanding of the factors influencing their QoL and mental health.

## Figures and Tables

**Table 1:** Sample characteristics.

Variable	Characteristics (n = 156)
Age	≤50 (n = 66); >50 (n = 89); Mean 50.64 (n = 155, SD ± 9.6, age range 28–73)
Sex	Male (n = 89); Female (n = 67)
Language for completing Questionnaire	English (n = 59); Arabic (n = 98)
Religion	Christians (n = 155); Other (n = 1)
Marital status	Living with a partner (n = 146); Not living with a partner (n = 10)
Employment status	Employed (n = 154); Unemployed (n = 2)
Ethnic group	Egyptian (n = 137); Other (n = 19)
Employment type	Professional and semi-professional (n = 83); Skilled and self-employed (n = 37)
Years of retirement	0–9 years (n = 12); 10–20+ years (n = 17); Note: Most of participants are still employed.
Reasons for retirement	Tired and age (n = 12); Illness (n = 11); Family reasons (n = 5); Note: Most of participants are still employed.

**Table 2:** Descriptive Statistics of 26 Likert scale questions.

	n	Minimum	Maximum	Mean	Std. Deviation
QoL 1 Material comforts home, food, conveniences, financial security	156	1	7	5.25	1.389
QoL 2 Health- being physically fit and vigorous	156	1	7	4.88	1.379
QoL 3 Relationship with parents, siblings and other relatives- communicating, visiting, helping	156	1	7	5.36	1.423
QoL 4 Having and rearing children	151	1	7	5.50	1.712
QoL 5 Close relationships with spouse or significant other	155	1	7	5.52	1.568
QoL 6 Close friends	156	1	7	5.21	1.481
QoL 7 Helping and encouraging others, volunteering, giving advice	156	1	7	5.12	1.474
QoL 8 Participating in organizations and public affairs	156	1	7	4.23	1.897
QoL 9 Learning-attending school, improving understanding, getting additional knowledge	155	1	7	4.90	1.567
QoL 10 Understanding yourself—knowing your assets and limitations—improving understanding—know what life is about	156	1	7	5.44	1.360
QoL 11 Work-job or in home	155	1	7	5.22	1.456
QoL 12 Expressing yourself creatively	156	1	7	5.06	1.390
QoL 13 Socializing-meeting other people, doing things, parties, etc.….	156	1	7	5.00	1.428
QoL 14 Reading, listening to music, or observing entertainment	156	1	7	5.09	1.398
QoL 15 Participating in active recreation	156	1	7	4.54	1.647
QoL 16 Independence, doing for yourself	156	1	7	5.38	1.487
K-10, 1 In the last four weeks, about how often did you feel tired out for no good reason?	156	1	6	2.75	1.189
K-10, 2 In the last four weeks About how often did you feel nervous?	156	1	5	2.61	1.081
K-10, 3 In the last four weeks about how often did you feel so nervous that nothing could calm you down?	156	1	5	2.11	1.087
K-10, 4 In the last four weeks about how often did you feel hopeless?	156	1	5	1.81	1.002
K-10, 5 In the last four weeks about how often did you feel restless or fidgety?	156	1	5	2.22	1.128
K-10, 6 In the last four weeks about how often did you feel so restless you could not sit still?	156	1	5	1.97	1.098
K-10, 7 In the last four weeks about how often did you feel depressed?	156	1	5	1.92	1.057
K-10, 8 In the last four weeks about how often did you feel that everything was an effort?	156	1	5	2.24	1.192
K-10, 9 In the last four weeks about how often did you feel so sad that nothing could cheer you up?	156	1	5	2.04	1.083
K-10, 10 In the last four weeks about how often did you feel worthless?	156	1	5	1.78	1.132

**Table 3:** Non-parametric correlation between all the questions regarding qualify of life and K-10.

	Mean, K10	Mean QoL	QoL 1	QoL 2	QoL 3	QoL 4	QoL 5	QoL 6	QoL 7	QoL 8	QoL 9	QoL 10	QoL 11	QoL 12	QoL 13	QoL 14	QoL 15	QoL 16	K10, 1	K-10, 2	K-10, 3	K-10, 4	K-10, 5	K-10, 6	K-10, 7	K-10, 8	K-10, 9	K-10, 10
Mean, K10	1.000																											
Mean Qol	−0.347 **	1.000																										
QoL 1	−0.373 **	0.599 **	1.000																									
QoL 2	−0.304 **	0.508 **	0.624 **	1.000																								
QoL 3	−0.311 **	0.573 **	0.503 **	0.462 **	1.000																							
QoL 4	−0.188 *	0.460 **	0.372 **	0.413 **	0.418 **	1.000																						
QoL 5	−0.300 **	0.627 **	0.562 **	0.330 **	0.400 **	0.433 **	1.000																					
QoL 6	−0.251 **	0.637 **	0.430 **	0.341 **	0.473 **	0.481 **	0.536 **	1.000																				
QoL 7	−0.176 *	0.676 **	0.377 **	0.375 **	0.443 **	0.299 **	0.316 **	0.532 **	1.000																			
QoL 8	−0.152	0.510 **	0.253 **	0.262 **	0.357 **	0.204 *	0.170 *	0.364 **	0.451 **	1.000																		
QoL 9	−0.144	0.577 **	0.335 **	0.335 **	0.333 **	0.219 **	0.357 **	0.403 **	0.368 **	0.381 **	1.000																	
QoL 10	−0.301 **	0.719 **	0.516 **	0.441 **	0.443 **	0.215 **	0.470 **	0.519 **	0.615 **	0.324 **	0.454 **	1.000																
QoL 11	−0.379 **	0.642 **	0.507 **	0.375 **	0.454 **	0.294 **	0.512 **	0.481 **	0.469 **	0.286 **	0.331 **	0.604 **	1.000															
QoL 12	−0.298 **	0.718 **	0.382 **	0.315 **	0.409 **	0.244 **	0.509 **	0.399 **	0.485 **	0.312 **	0.507 **	0.639 **	0.627 **	1.000														
QoL 13	−0.256 **	0.701 **	0.427 **	0.396 **	0.528 **	0.432 **	0.504 **	0.517 **	0.505 **	0.384 **	0.468 **	0.478 **	0.471 **	0.630 **	1.000													
QoL 14	−0.217 **	0.638 **	0.427 **	0.416 **	0.312 **	0.385 **	0.498 **	0.388 **	0.382 **	0.194 *	0.397 **	0.390 **	0.455 **	0.493 **	0.493 **	1.000												
QoL 15	−0.176 *	0.655 **	0.319 **	0.329 **	0.290 **	0.241 **	0.320 **	0.448 **	0.450 **	0.316 **	0.464 **	0.389 **	0.375 **	0.533 **	0.534 **	0.610 **	1.000											
QoL 16	−0.122	0.577 **	0.341 **	0.244 **	0.378 **	0.250 **	0.324 **	0.274 **	0.355 **	0.138	0.389 **	0.465 **	0.541 **	0.506 **	0.382 **	0.416 **	0.337 **	1.000										
K10, 1	0.619 **	−0.098	−0.135	−0.107	−0.088	−0.008	−0.117	−0.067	−0.013	−0.086	−0.018	−0.085	−0.160 *	−0.082	−0.035	−0.060	−0.034	0.057	1.000									
K-10, 2	0.720 **	−0.195 *	−0.133	−0.110	−0.089	0.024	−0.061	−0.162 *	−0.111	−0.216 **	−0.067	−0.156	−0.229 **	−0.126	−0.150	−0.073	−0.121	0.007	0.489 **	1.000								
K-10, 3	0.791 **	−0.301 **	−0.316 **	−0.287 **	−0.176 *	−0.141	−0.210 **	−0.244 **	−0.168 *	−0.103	−0.144	−0.287 **	−0.289 **	−0.237 **	−0.205 *	−0.237 **	−0.111	−0.113	0.419 **	0.631 **	1.000							
K-10, 4	0.717 **	−0.362 **	−0.323 **	−0.306 **	−0.282 **	−0.208 *	−0.328 **	−0.228 **	−0.215 **	−0.145	−0.122	−0.345 **	−0.356 **	−0.283 **	−0.281 **	−0.206 **	−0.079	−0.212 **	0.370 **	0.439 **	0.590 **	1.000						
K-10, 5	0.776 **	−0.191 *	−0.255 **	−0.249 **	−0.221 **	−0.185 *	−0.279 **	−0.176 *	−0.081	−0.017	0.012	−0.190 *	−0.314 **	−0.166 *	−0.163 *	−0.271 **	−0.080	−0.048	0.391 **	0.523 **	0.593 **	0.500 **	1.000					
K-10, 6	0.778 **	−0.283 **	−0.302 **	−0.224 **	−0.265 **	−0.144	−0.235 **	−0.121	−0.080	−0.094	−0.074	−0.199 *	−0.329 **	−0.256 **	−0.209 **	−0.212 **	−0.166 *	−0.124	0.349 **	0.443 **	0.543 **	0.518 **	0.669 **	1.000				
K-10, 7	0.745 **	−0.339 **	−0.395 **	−0.307 **	−0.363 **	−0.173 *	−0.270 **	−0.186 *	−0.256 **	−0.086	−0.054	−0.301 **	−0.330 **	−0.271 **	−0.312 **	−0.166 *	−0.149	−0.133	0.310 **	0.444 **	0.536 **	0.603 **	0.518 **	0.597 **	1.000			
K-10, 8	0.773 **	−0.306 **	−0.307 **	−0.301 **	−0.376 **	−0.140	−0.197 *	−0.174 *	−0.207 **	−0.090	−0.241 **	−0.289 **	−0.304 **	−0.316 **	−0.289 **	−0.186 *	−0.192 *	−0.178 *	0.394 **	0.456 **	0.504 **	0.526 **	0.544 **	0.604 **	0.700 **	1.000		
K-10, 9	0.845 **	−0.306 **	−0.376 **	−0.283 **	−0.286 **	−0.196 *	−0.293 **	−0.270 **	−0.092	−0.170 *	−0.189 *	−0.237 **	−0.325 **	−0.251 **	−0.208 **	−0.138	−0.153	−0.127	0.388 **	0.575 **	0.620 **	0.621 **	0.630 **	0.683 **	0.607 **	0.639 **	1.000	
K-10, 10	0.716 **	−0.315 **	−0.326 **	−0.220 **	−0.310 **	−0.198 *	−0.252 **	−0.248 **	−0.130	−0.063	−0.170 *	−0.227 **	−0.313 **	−0.327 **	−0.252 **	−0.157 *	−0.170 *	−0.240 **	0.249 **	0.433 **	0.554 **	0.582 **	0.433 **	0.614 **	0.599 **	0.613 **	0.746 **	1.000

Question descriptions see Table 2. Significance level: * *p* < 0.05, ** *p* < 0.01.

**Table 4:** Partial correlation between all the questions regarding qualify of life (QoL) and Kessler Psychological Distress Scale (K10).

	Mean, K10	Mean QoL	QoL 1	QoL 2	QoL 3	QoL 4	QoL 5	QoL 6	QoL 7	QoL 8	QoL 9	QoL 10	QoL 11	QoL 12	QoL 13	QoL 14	QoL 15	QoL 16	K10, 1	K-10, 2	K-10, 3	K-10, 4	K-10, 5	K-10, 6	K-10, 7	K-10, 8	K-10, 9	K-10, 10
Mean, K10	1.000	−0.347 **	−0.328 **	−0.338 **	−0.304 **	−0.156	−0.241 **	−0.180 *	−0.201 *	−0.132	−0.108	−0.299 **	−0.428 **	−0.332 **	−0.258 **	−0.208 *	−0.148	−0.122	0.575 **	0.734 **	0.792 **	0.764 **	0.751 **	0.823 **	0.809 **	0.795 **	0.871 **	0.796 **
Mean Qol		1.000	0.654 **	0.616 **	0.563 **	0.455 **	0.643 **	0.649 **	0.675 **	0.485 **	0.591 **	0.743 **	0.658 **	0.755 **	0.718 **	0.659 **	0.657 **	0.533 **	−0.088	−0.181 *	−0.301 **	−0.373 **	−0.203 *	−0.253 **	−0.364 **	−0.307 **	−0.327 **	−0.296 **
QoL 1			1.000	0.668 **	0.470 **	0.324 **	0.586 **	0.404 **	0.403 **	0.215 **	0.285 **	0.536 **	0.454 **	0.406 **	0.436 **	0.452 **	0.324 **	0.298 **	−0.130	−0.101	−0.283 **	−0.288 **	−0.234 **	−0.272 **	−0.387 **	−0.272 **	−0.310 **	−0.265 **
QoL 2				1.000	0.443 **	0.381 **	0.410 **	0.310 **	0.383 **	0.266 **	0.248 **	0.500 **	0.403 **	0.409 **	0.431 **	0.433 **	0.381 **	0.238 **	−0.098	−0.098	−0.320 **	−0.328 **	−0.247 **	−0.268 **	−0.359 **	−0.302 **	−0.334 **	−0.266 **
QoL 3					1.000	0.343 **	0.399 **	0.391 **	0.373 **	0.308 **	0.259 **	0.391 **	0.376 **	0.379 **	0.466 **	0.286 **	0.230 **	0.309 **	−0.141	−0.071	−0.138	−0.301 **	−0.216 **	−0.239 **	−0.296 **	−0.392 **	−0.283 **	−0.261 **
QoL 4						1.000	0.358 **	0.385 **	0.288 **	0.160	0.154	0.233 **	0.252 **	0.242 **	0.370 **	0.324 **	0.189 *	0.230 **	−0.068	−0.013	−0.111	−0.164 *	−0.187 *	−0.110	−0.148	−0.111	−0.167 *	−0.129
QoL 5							1.000	0.470 **	0.352 **	0.157	0.255 **	0.468 **	0.471 **	0.452 **	0.497 **	0.513 **	0.294 **	0.250 **	−0.087	−0.040	−0.200 *	−0.266 **	−0.255 **	−0.203 *	−0.249 **	−0.163 *	−0.221 **	−0.186 *
QoL 6								1.000	0.541 **	0.329 **	0.388 **	0.507 **	0.474 **	0.359 **	0.440 **	0.367 **	0.436 **	0.224 **	−0.047	−0.151	−0.224 **	−0.184 *	−0.128	−0.067	−0.147	−0.097	−0.194 *	−0.160
QoL 7									1.000	0.452 **	0.348 **	0.589 **	0.436 **	0.476 **	0.466 **	0.401 **	0.427 **	0.266 **	−0.054	−0.124	−0.173 *	−0.274 **	−0.138	−0.082	−0.294 **	−0.173 *	−0.130	−0.126
QoL 8										1.000	0.390 **	0.310 **	0.300 **	0.285 **	0.343 **	0.142	0.302 **	0.090	−0.086	−0.233 **	−0.096	−0.149	−0.002	−0.082	−0.075	−0.086	−0.179 *	−0.041
QoL 9											1.000	0.430 **	0.334 **	0.543 **	0.419 **	0.375 **	0.485 **	0.316 **	0.032	−0.058	−0.118	−0.074	0.056	−0.063	−0.041	−0.196 *	−0.190 *	−0.178 *
QoL 10												1.000	0.548 **	0.685 **	0.526 **	0.394 **	0.412 **	0.366 **	−0.077	−0.153	−0.302 **	−0.345 **	−0.206 *	−0.205 *	−0.351 **	−0.247 **	−0.241 **	−0.198 *
QoL 11													1.000	0.562 **	0.439 **	0.410 **	0.339 **	0.429 **	−0.239 **	−0.315 **	−0.368 **	−0.407 **	−0.385 **	−0.387 **	−0.317 **	−0.282 **	−0.351 **	−0.258 **
QoL 12														1.000	0.638 **	0.501 **	0.522 **	0.508 **	−0.082	−0.173 *	−0.277 **	−0.348 **	−0.206 *	−0.272 **	−0.301 **	−0.325 **	−0.270 **	−0.314 **
QoL 13															1.000	0.513 **	0.544 **	0.318 **	−0.033	−0.145	−0.194 *	−0.266 **	−0.161	−0.200 *	−0.295 **	−0.277 **	−0.225 **	−0.203 *
QoL 14																1.000	0.591 **	0.450 **	−0.062	−0.057	−0.212 **	−0.187 *	−0.236 **	−0.183 *	−0.187 *	−0.175 *	−0.147	−0.164 *
QoL 15																	1.000	0.290 **	−0.059	−0.128	−0.113	−0.094	−0.055	−0.077	−0.139	−0.196 *	−0.153	−0.125
QoL 16																		1.000	0.048	−0.015	−0.067	−0.234 **	−0.023	−0.087	−0.121	−0.144	−0.121	−0.196 *
K10, 1																			1.000	0.487 **	0.394 **	0.373 **	0.395 **	0.412 **	0.318 **	0.381 **	0.379 **	0.244 **
K-10, 2																				1.000	0.671 **	0.482 **	0.507 **	0.496 **	0.479 **	0.458 **	0.600 **	0.480 **
K-10, 3																					1.000	0.602 **	0.572 **	0.578 **	0.592 **	0.497 **	0.636 **	0.585 **
K-10, 4																						1.000	0.482 **	0.539 **	0.624 **	0.543 **	0.659 **	0.635 **
K-10, 5																							1.000	0.712 **	0.543 **	0.517 **	0.616 **	0.444 **
K-10, 6																								1.000	0.635 **	0.639 **	0.701 **	0.634 **
K-10, 7																									1.000	0.713 **	0.675 **	0.675 **
K-10, 8																										1.000	0.683 **	0.675 **
K-10, 9																											1.000	0.777 **
K-10, 10																												1.000

Question descriptions see Table 2. Significance level: * *p* < 0.05, ** *p* < 0.01.

**Table 5:** Logistic regression to indicate the predicting effects of Quality of Life and -K10 on the four demographic factors respectively.

	Quality of Life (QoL)					Kessler Psychological Distress Scale (K10)				
	Chi-Square	df	*p*	Nagelkerke R^2^	N	Chi-Square	df	*p*	Nagelkerke R^2^	N
Marital status	6.723	16	0.978	0.113	149	12.161	10	0.274	0.198	155
Age (≤50 years vs. >50 years)	17.524	16	0.353	0.148	150	14.858	10	0.137	0.122	156
Employment status (full time vs. part time and retired)	13.197	16	0.658	0.112	150	7.326	10	0.694	0.061	156
Employment type (Professional and semi-professional vs. skilled and sale)	27.700	16	0.034	0.292	116	10.458	10	0.401	0.114	121
Gender (male vs. female)	18.849	16	0.277	0.158	150	5.216	10	0.876	0.044	156
Marital status	6.723	16	0.978	0.113	149	12.161	10	0.274	0.198	155
Language for completing survey questions (English vs. Arabic)	22.929	16	0.116	0.342	150	14.386	10	0.156	0.120	156

## Data Availability

The data presented in this study are available on request from the corresponding author due to privacy reasons.
